# Supplementary material for: A DNA Prime Immuno-Potentiates a Modified Live Vaccine against the Porcine Reproductive and Respiratory Syndrome Virus but Does Not Improve Heterologous Protection
Source: Viruses. 2019 Jun 25;11(6):576. doi: 10.3390/v11060576 (PMC6631340; doi:10.3390/v11060576)
Supplement: Supplementary file 1 [file viruses-11-00576-s001.zip › viruses-520231-supplementary/Supplementary Figures.pdf]

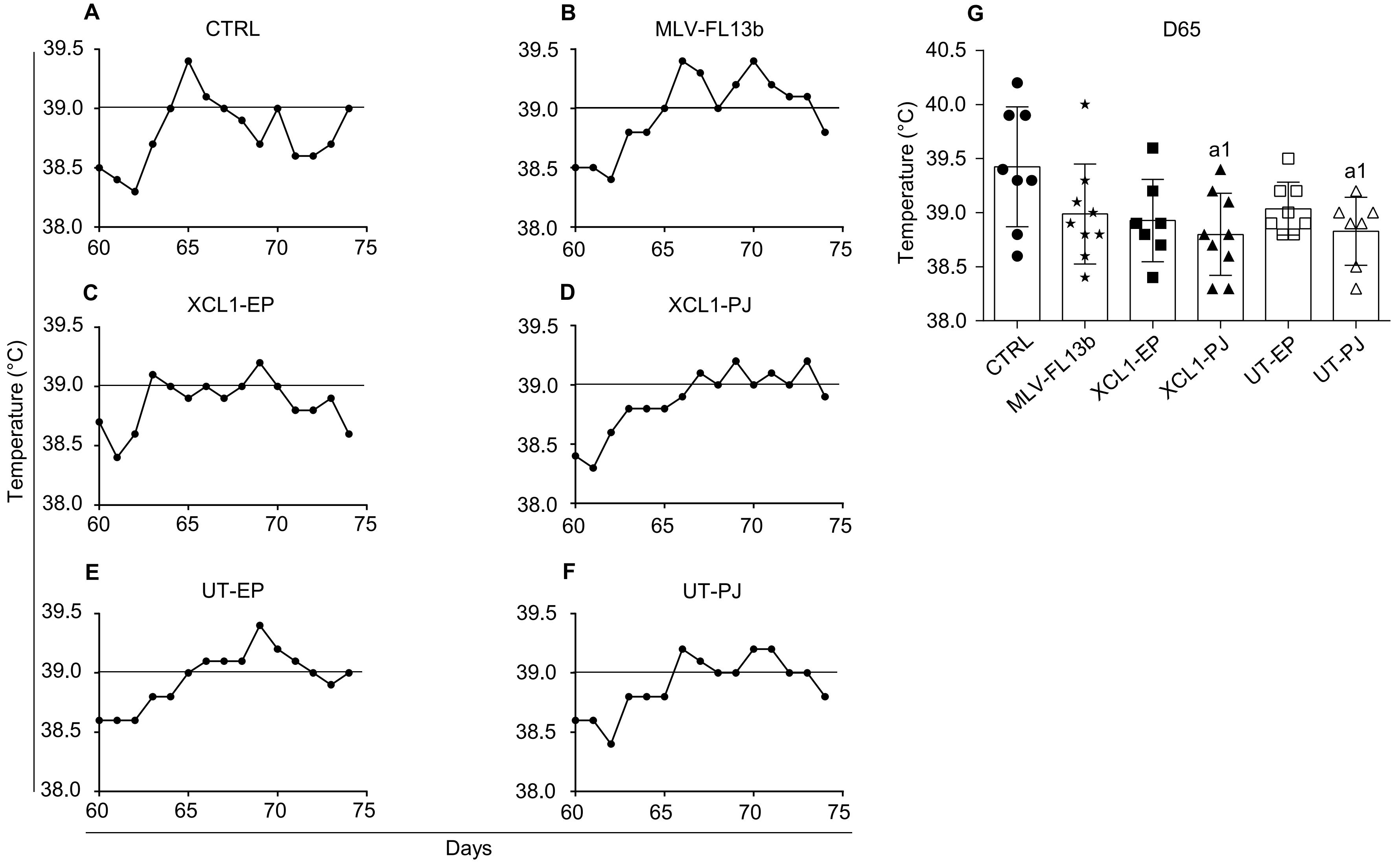

Supplementary figure 1

Monitoring of body temperature in pigs challenged with FL07. **A-F.** The temperature of the vaccinated pigs (one group per panel) was monitored with subcutaneous sensor chips and the mean values from D60 to 75 are reported per group. **G**. The individual body temperatures are reported for each group at D65 (mean as a box, ± sem).

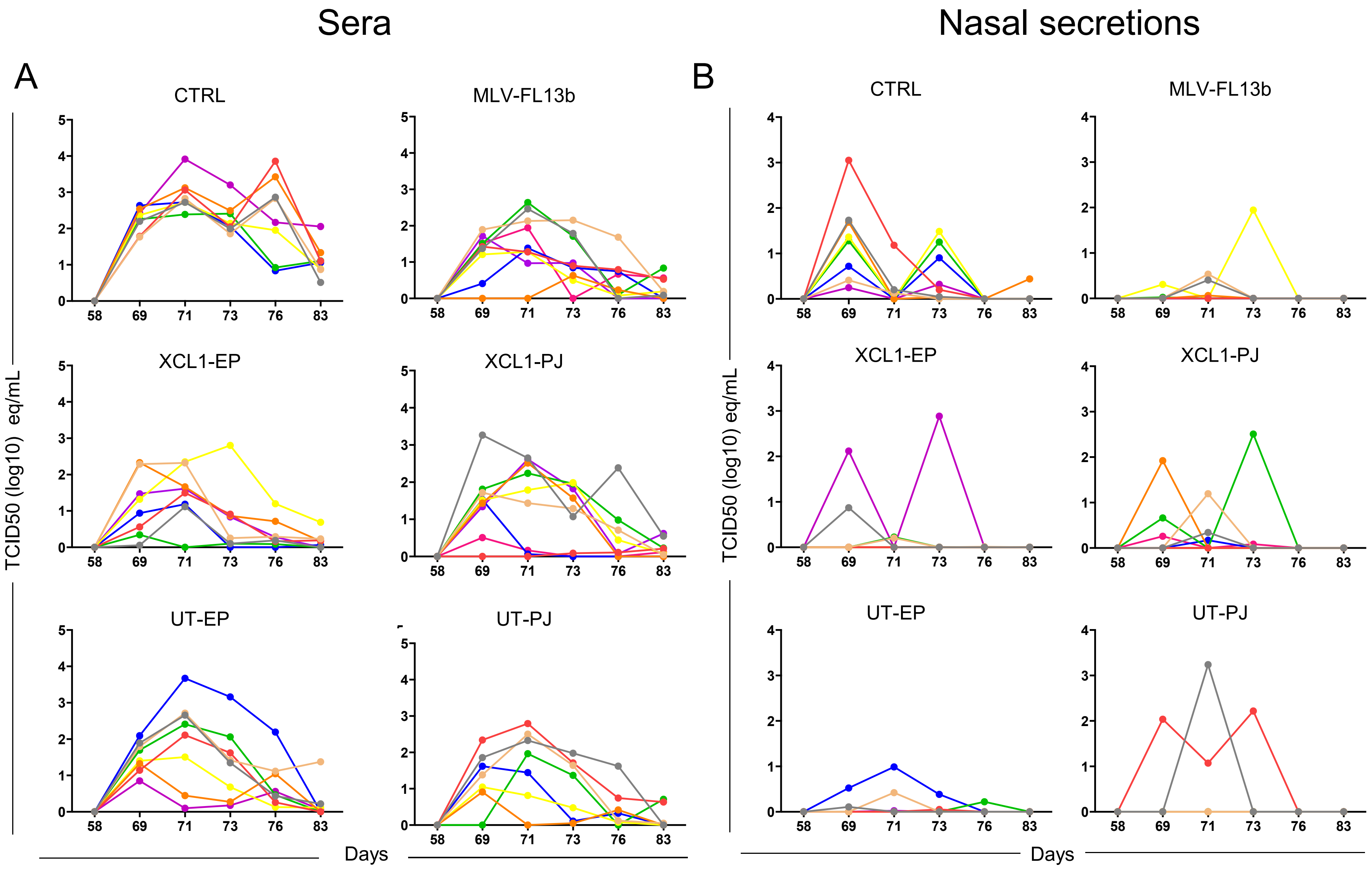

Supplementary figure 2
Detection over time of FL07 RNA in serum (A) and nasal secretions (B) of unvaccinated, MLV-13b and DNA+MLV-FL13b vaccinated pigs. The vaccinated pigs were subjected to FL07 challenge on D63 and the FL07 virus was selectively detected in sera and nasal swab fluids with specific qRT-PCR on D58, 69, 71, 73, 76 and 83. The $TCID_{50}$eq (log10)/ml are indicated at each time point for each pig of the 6 groups. The limit of detection is calculated as being 0.2 $TCID_{50}$eq/ml.

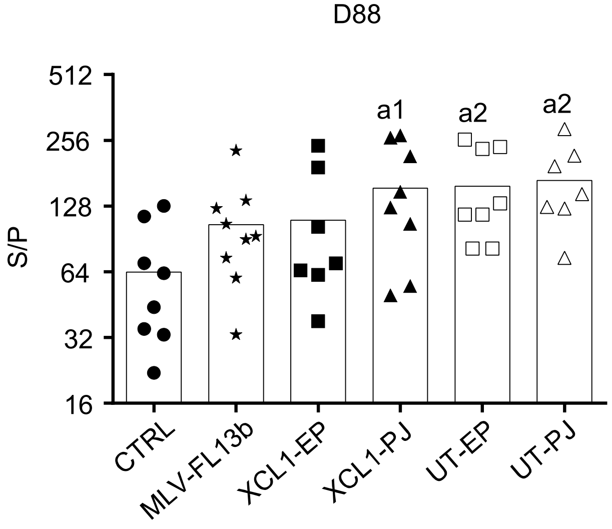

Supplementary figure 3

Effect of the FL07 heterologous challenge on anti-Env Abs in MLV-FL13b and DNA+MLV vaccinated pigs. Sera collected at D88 were analyzed at a 1:20 dilution with the ELISA-Env. Statistically significant differences between 2 groups were calculated using the Mann Whitney non-parametric test. Number 1 corresponds to $p < 0.05$, 2 to $p < 0.01$.
