# Supplementary material for: A DNA Prime Immuno-Potentiates a Modified Live Vaccine against the Porcine Reproductive and Respiratory Syndrome Virus but Does Not Improve Heterologous Protection
Source: Viruses. 2019 Jun 25;11(6):576. doi: 10.3390/v11060576 (PMC6631340; doi:10.3390/v11060576)
Supplement: Supplementary file 1 [file viruses-11-00576-s001.zip › viruses-520231-supplementary/Supplementary Table 1.pdf]

GP4GP5M chimera
H- MAATILFLLAGAQHFMVSEA -OH
H- LAGAQHFMVSEAFACKPCFS -OH
H- VSEAFACKPCFSTHLSDIKT -OH
H- PCFSTHLSDIKTNTTAAAGF -OH
H- DIKTNTTAAAGFMVLQNINC -OH
H- AAGFMVLQNINCPQFHRAST -OH
H- NINCPQFHRASTTSSSPLRK -OH
H- RASTTSSSPLRKSPQCREAV -OH
H- PLRKSPQCREAVGTPQYITI -OH
H- REAVGTPQYITIVANVTDES -OH
H- YITIVANVTDESYLYNADLL -OH
H- TDESYLYNADLLMLSACLFY -OH
H- ADLLMLSACLFYASEMSEKG -OH
H- CLFYASEMSEKGFKVIFGNF -OH
H- SEKGFKVIFGNFADGNGDSS -OH
H- FGNFADGNGDSSTYQYIYNL -OH
H- GDSSTYQYIYNLTICELNGT -OH
H- IYNLTICELNGTAWLSDKFY -OH
H- LNGTAWLSDKFYWAVAIRAA -OH
H- DKFYWAVAIRAAKNCMACRY -OH
H- IRAAKNCMACRYARTRFTNF -OH
H- ACRYARTRFTNFIVDDRGGV -OH
H- FTNFIVDDRGGVHRWKSPIV -OH
H- RGGVHRWKSPIVVEKLGKAE -OH
H- SPIVVEKLGKAEVGDALVTI -OH
H- GKAEVGDALVTIKHVVIEGV -OH
H- LVTIKHVVIEGVKAQPLTRT -OH
H- IEGVKAQPLTRTTAEQWQAA -OH
H- LTRTTAEQWQAAGLDDFCYD -OH
H- WQAAGLDDFCYDSTAVQKLS -OH
H- FCYDSTAVQKLSRCRLCCLG -OH
H- QKLSRCRLCCLGRRYILAPA -OH
H- CCLGRRYILAPAHHVESAAG -OH
H- LAPAHHVESAAGLHPIPASG -OH
H- SAAGLHPIPASGNQAYAVRK -OH
H- PASGNQAYAVRKPGLTSVNG -OH
H- AVRKPGLTSVNGTLVPGLRG -OH
H- SVNGTLVPGLRGLVLGGKRA -OH
H- GLRGLVLGGKRAVKRGMVNL -OH
H- VLGGKRAVKRGMVNLVKYGR -OH

Nucleoprotein N
H- MAGKTQRQNRNKNPAPMGNG -OH
H- NRNKNPAPMGNGQSVNQLCQ -OH
H- MGNGQSVNQLCQLLGSMLKS -OH
H- QLCQLLGSMLKSQRQQSRGG -OH
H- MLKSQRQQSRGGQVKKKKPE -OH
H- SRGGQVKKKKPEKPHFPLAA -OH
H- KKPEKPHFPLAAEDDVRHHL -OH
H- PLAAEDDVRHHLTQAERSLC -OH
H- RHHLTQAERSLCLQSIQTAF -OH
H- RSLCLQSIQTAFNQGAGTAS -OH
H- QTAFNQGAGTASLSSSGKVG -OH
H- GTASLSSSGKVGFQVEFMLP -OH
H- GKVGFQVEFMLPVTHTVRLI -OH
H- FMLPVTHTVRLIRVTSTSAS -OH
H- VTHTVRLIRVTSTSASQGVN -OH

NSP1β
H- MSGTFSRCMCTPAARVFWNA -OH
H- MCTPAARVFWNAGQVYCTRC -OH
H- FWNAGQVYCTRCLSARSLLP -OH
H- CTRCLSARSLLPLELQDDDL -OH
H- SLLPLELQDDDLGAIGLFHK -OH
H- DDDLGAIGLFHKPKDKLRWR -OH
H- LFHKPKDKLRWRVPVGIPLV -OH

H- LRWRVPVGIPLVECSPSGCC -OH
H- IPLVECSPSGCCWLSAIFPL -OH
H- SGCCWLSAIFPLARMTSGNH -OH
H- IFPLARMTSGNHNFLQRLVK -OH
H- SGNHNFLQRLVKVAEVLYRD -OH
H- RLVKVAEVLYRDGCLTPRHL -OH
H- LYRDGCLTPRHLRELQVYER -OH
H- PRHLRELQVYERGCDWYPIT -OH
H- VYERGCDWYPITGPVPGMGM -OH
H- YPITGPVPGMGMYANSMHVS -OH
H- GMGMYANSMHVSDRPFPGAT -OH
H- MHVSDRPFPGATHVLTNSPL -OH
H- PGATHVLTNSPLPQQACRQP -OH
H- NSPLPQQACRQPFCPFEEAH -OH
H- CRQPFCPFEEAHSDVYKWKK -OH
H- EEAHSDVYKWKKFVIFTDSS -OH
H- KWKKFVIFTDSSPNGRSRMM -OH
H- TDSSPNGRSRMMWMPESGDS -OH
H- SRMMWMPESGDSANLEELPL -OH
H- SGDSANLEELPLELERQVEI -OH
H- ELPLELERQVEILVRSFPAH -OH
H- QVEILVRSFPAHHPVDLADW -OH
H- FPAHHPVDLADWELTESPEH -OH
H- LADWELTESPEHGFSFGTSH -OH
H- SPEHGFSFGTSHHCGYLAQH -OH
H- GTSHHCGYLAQHPYGFDGKC -OH
H- LAQHPYGFDGKCWLSCFLDL -OH
H- DGKCWLSCFLDLSTKVLRHE -OH
H- FLDLSTKVLRHEEYLASAFG -OH
H- LRHEEYLASAFGYQTRWGVP -OH
H- SAFGYQTRWGVPGKYLQRRL -OH
H- WGVPGKYLQRRLQINGVRAV -OH
H- QRRLQINGVRAVVDPDGPIH -OH
H- VRAVVDPDGPIHVEALSCPQ -OH
H- GPIHVEALSCPQSWIRHLTL -OH
H- SCPQSWIRHLTLDDDATPGF -OH
H- HLTLDDDATPGFVRLMSLRI -OH
H- TPGFVRLMSLRIIPNTEPTT -OH
H- SLRIIPNTEPTTLQIFRFGT -OH
H- IPNTEPTTLQIFRFGTHKWY -OH

RdRp pool1

H- MATGFKLLAASGLTRCGRGG -OH
H- AASGLTRCGRGGLVVTETAV -OH
H- GRGGLVVTETAVKIVKYHSR -OH
H- ETAVKIVKYHSRTFTLGPLD -OH
H- YHSRTFTLGPLDLKVTSEVE -OH
H- GPLDLKVTSEVEVKKSTEQG -OH
H- SEVEVKKSTEQGHAVVANLC -OH
H- TEQGHAVVANLCSGVVLMRP -OH
H- ANLCSGVVLMRPHPPSLVDV -OH
H- LMRPHPPSLVDVILKPGLDT -OH
H- LVDVILKPGLDTTPGIQPGH -OH
H- GLDTTPGIQPGHGAGNMGVD -OH
H- QPGHGAGNMGVDGTIWDFET -OH
H- MGVDGTIWDFETAPTRAELE -OH
H- DFETAPTRAELELSKQIIQA -OH
H- AELELSKQIIQACEIRRGDA -OH
H- IIQACEIRRGDAPNLQLPYK -OH
H- RGDAPNLQLPYKLYPVRGDP -OH
H- LPYKLYPVRGDPERKEGRLI -OH
H- RGDPERKEGRLINTRFGDLP -OH
H- GRLINTRFGDLPYKTPQDTG -OH
H- GDLPYKTPQDTGSAIHAACC -OH
H- QDTGSAIHAACCLNPNGAPV -OH
H- AACCLNPNGAPVSDGKSVLG -OH

H- GAPVSDGKSVLGTTLQHGFE -OH
H- SVLGTTLQHGFELYVPTVPY -OH
H- HGFELYVPTVPYSVMEYLDS -OH
H- TVPYSVMEYLDSRPDTPLMC -OH
H- YLDSRPDTPLMCTKHGTSRA -OH
H- PLMCTKHGTSRAATEDLQKY -OH
H- TSRAATEDLQKYDLSTQGFV -OH
H- LQKYDLSTQGFVLPGVLRLV -OH
H- QGFVLPGVLRLVRRFIFGHI -OH
H- LRLVRRFIFGHIGKAPPLFL -OH
H- FGHIGKAPPLFLPSTYPAKN -OH
H- PLFLPSTYPAKNSMAGINGQ -OH
H- PAKNSMAGINGQRFPTKDVQ -OH
H- INGQRFPTKDVQSIPEVDEM -OH
H- KDVQSIPEVDEMCARAVKEN -OH
H- VDEMCARAVKENWQTVTPCT –OH

RdRp pool2
H- VKENWQTVTPCTLKKQYCSK -OH
H- TPCTLKKQYCSKPKTRTILG -OH
H- YCSKPKTRTILGTNNFIALA -OH
H- TILGTNNFIALAHRSALSGV -OH
H- IALAHRSALSGVTQAFMKKA -OH
H- LSGVTQAFMKKAWRSPIALG -OH
H- MKKAWRSPIALGKNKFKELH -OH
H- IALGKNKFKELHCTVAGRCL -OH
H- KELHCTVAGRCLEADLASCD -OH
H- GRCLEADLASCDRSTPAIVR -OH
H- ASCDRSTPAIVRWFTAHLLY -OH
H- AIVRWFTAHLLYELAGCEEY -OH
H- HLLYELAGCEEYLPSYVLNC -OH
H- CEEYLPSYVLNCCHDLVATQ -OH
H- VLNCCHDLVATQDGAFTKRG -OH
H- VATQDGAFTKRGGLSSGDPV -OH
H- TKRGGLSSGDPVTSVSNTVY -OH
H- GDPVTSVSNTVYSLIIYAQH -OH
H- NTVYSLIIYAQHMVLSALKM -OH
H- YAQHMVLSALKMGHEIGLKF -OH
H- ALKMGHEIGLKFLEDQLKFE -OH
H- GLKFLEDQLKFEDLLEIQPM -OH
H- LKFEDLLEIQPMLVYSDDLV -OH
H- IQPMLVYSDDLVLYAEQPTF -OH
H- DDLVLYAEQPTFPNYHWWVE -OH
H- QPTFPNYHWWVEHLDLMLGF -OH
H- WWVEHLDLMLGFKTDPKKTV -OH
H- MLGFKTDPKKTVITDKPSFL -OH
H- KKTVITDKPSFLGCKIEAGR -OH
H- PSFLGCKIEAGRQLVPNRDR -OH
H- EAGRQLVPNRDRILAALAYH -OH
H- NRDRILAALAYHMKAQNASE -OH
H- LAYHMKAQNASEYYASAAAI -OH
H- NASEYYASAAAILMDSCACI -OH
H- AAAILMDSCACIDYDPEWYE -OH
H- CACIDYDPEWYEDLICGIAR -OH
H- EWYEDLICGIARCARQDGYS -OH
H- GIARCARQDGYSFPGPPFFM -OH
H- DGYSFPGPPFFMSMWERLKS -OH
H- SFPGPPFFMSMWERLKSHNE -OH

Control peptide
H- IKDFHVYFRESRDALWKGPG -OH
